# Achieving EQE of 16,700% in P3HT:PC_71_BM based photodetectors by trap-assisted photomultiplication

**DOI:** 10.1038/srep09181

**Published:** 2015-03-17

**Authors:** Lingliang Li, Fujun Zhang, Jian Wang, Qiaoshi An, Qianqian Sun, Wenbin Wang, Jian Zhang, Feng Teng

**Affiliations:** 1 Key Laboratory of Luminescence and Optical Information, Ministry of Education, Beijing Jiaotong University, Beijing 100044, People's Republic of China; 2 State Key Laboratory of Catalysis, Dalian institute of Chemical Physics, Chinese Academy of Sciences, Dalian 116023, People's Republic of China

## Abstract

We report a trap-assisted photomultiplication (PM) phenomenon in solution-processed polymer photodetectors (PPDs) based on P3HT:PC_71_BM as the active layer, the maximum EQE of 16,700% is obtained for the PPDs with PC_71_BM doping weight ratio of 1%. The PM phenomenon is attributed to the enhanced hole tunneling injection assisted by trapped electrons in PC_71_BM near Al cathode, which can be demonstrated by the transient photocurrent curves and EQE spectra of PPDs with different PC_71_BM doping ratios. The positive effect of trapped electrons in PC_71_BM near Al cathode on the hole tunneling injection is further confirmed by the simulated optical field and exciton generation rate distributions in the active layer and the EQE spectra of PPDs with Al(1)/P3HT:PC_71_BM(100:1)/Al(2) device structure under forward and reverse biases. This discovery may open a new road for organic materials to be used in highly sensitive photodetectors while preserving the advantages of organic materials.

Organic/polymer optoelectronic devices have been rapidly developed in the past years due to their advantages such as flexibility, low cost and simple fabrication techniques^1^,^2^,^3^. Even more interesting is that direct optical transition is allowed in organic/polymer semiconductors, which makes them promising candidates for light sensitive applications, such as solar cells and photodetectors^4^,^5^. Up to now, most of reported organic photodetectors exhibit external quantum efficiency (EQE) values lower than unit, which obeys the photovoltaic working mechanism^6^,^7^. Therefore, potential application of photodiode type photodetectors is limited by the low EQE. Yokoyama's group firstly reported a large photomultiplication (PM) phenomenon based on organic pigment layers simply sandwiched by two electrodes^8^,^9^. This phenomenon was reasonably interpreted in terms of electron tunneling injection triggered by the accumulation of photogenerated holes trapped near the interface between organic layer and metal electrode. Further investigations reveal that the interfacial traps are attributed to the poor or even nonexistent contact between electrode and organic layer because the evaporated metal electrode cannot follow the high surface roughness of organic layer^10^,^11^,^12^,^13^. Recently, Huang's group reported solution processed polymer/inorganic hybrid photodetectors exhibiting peak EQE values of 245,300% and 340,600% for ZnO nanoparticles doped poly-vinylcarbazole (PVK) and poly(3-hexylthiophene) (P3HT) as the active layers, respectively^14^. Sargent's group also reported a series of high performance solution processed photodetectors based on environmentally friendly sulfide nanocrystals (colloidal quantum dots) which exhibit high gain combined with high response speed^15^,^16^,^17^. Yang's group reported nanoparticle-assisted high EQE of 8,000% by additionally doping inorganic trap material in composites of polymer and fullerene^18^. Chen and Chuang et al. also reported high EQE values of 5,500% and 7,000% for photodetectors based on bulk heterojunction layers containing three or four kinds of organic materials^19^,^20^. For better understanding of the previous works on PM phenomenon based on organic or organic/inorganic hybrid materials, the schematic micro morphology of active layers for previous reported PM type photodetectors are shown in Figure S1.

As we know, P3HT and fullerene derivatives ([6,6]phenyl-C_71_-butyric acid methyl ester (PC_71_BM) and [6,6]phenyl-C_61_-butyric acid methyl ester (PC_61_BM)) are the most-prominent reported electron donor and acceptor materials in polymer solar cells (PSCs). To the best of our knowledge, the peak EQE values of reported photodetectors and PSCs based on P3HT:PCBM as the active layers are lower than 80%, which obeys the photovoltaic process^21^,^22^. The weight ratios of P3HT to PCBM are in the range from 1:0.8 to 1:1.2 to form bi-continuous interpenetrating network for better charge carrier transport in the active layer^23^,^24^. When PCBM doping weight ratio is rather low, photogenerated electrons are trapped in PCBM due to the absence of continuous electron transport channel. The existence of charge traps in active layer was considered to reduce the EQE of photodiode type photodetectors. In this paper, a relatively high EQE of 16,700% was obtained in polymer photodetectors (PPDs) based on P3HT:PCBM (100:1) as the active layers, which is among the highest reported values for photodetectors prepared from solely polymer/organic materials.

A series of solution-processed PPDs were fabricated with P3HT:PC_71_BM as the active layers. The only difference among these PPDs is the P3HT:PC_71_BM weight ratios ranging from 100:1 to 1:1 (device A, 100:1; device B, 100:4; device C, 100:15; device D, 100:50; device E, 1:1). Dark current density versus voltage (*J_d_-V*) curves of all the PPDs are shown in Figure 1a. It is known that the *J_d_* of devices is codetermined by charge carrier transport in the active layer and charge carrier injection barriers from electrodes to the active layer. The *J_d_* variation of devices can be understood from the energy levels of the used materials, as shown in the inserted image of Figure 1a. The barriers for electron injection from ITO onto the lowest unoccupied molecular orbital (LUMO) of P3HT and PC_71_BM are ~1.7 eV and 0.4 eV, respectively. Meanwhile, the barriers for hole injection from Al onto the highest occupied molecular orbital (HOMO) of P3HT and PC_71_BM are ~0.9 eV and 1.9 eV, respectively.

For devices D and E with relatively high PC_71_BM doping weight ratios, more electrons can be easily injected from ITO onto the LUMO of PC_71_BM due to the small injection barrier of ~0.4 eV and large contact interface between ITO and PC_71_BM. The injected electrons can be efficiently transported in the active layer along the continous electron transport channels due to high PC_71_BM doping ratios. Therefore, devices D and E have relatively large dark current and a limited withstand reverse bias lower than 2 V. For devices A, B and C with PC_71_BM doping weight ratios lower than 15%, electron injection from ITO onto the LUMO of PC_71_BM is limited due to the less contact interface between ITO and PC_71_BM, resulting in the relatively low dark current and high withstand reverse bias up to 19 V. In order to investigate the photoresponse of all the PPDs with different PC_71_BM doping weight ratios, EQE spectra of all the PPDs were measured under the given reverse biases and are shown in Figure 1b.

It is apparent that the EQE spectral shape of devices A, B and C with relatively low PC_71_BM doping weight ratios (≤15%) is distinctly different from that of devices D and E, as shown in Figure 1b. The EQE values of devices A, B and C are much larger than 100% in the spectral range from about 350 nm to 650 nm. However, the EQE values of devices D and E are lower than 100% in the whole spectral range, which well accords with the previously reported P3HT:PC_71_BM based PSCs^5^,^25^. Therefore, the PPDs with different PC_71_BM doping weight ratios can be classified as two different types: photodiode type PPDs with EQE lower than 100% and PM type PPDs with EQE higher than 100%.

According to the energy levels of PC_71_BM and P3HT, the isolated PC_71_BM aggregations in the blend films can be considered as electron traps due to the energy barrier of ~1.3 eV between the LUMOs of P3HT and PC_71_BM. The electrons trapped in the PC_71_BM near Al cathode can build up a Coulomb field (i.e., energy level curved) to assist hole tunneling injection from Al cathode onto the HOMO of P3HT under reverse bias. Holes will be continuously injected from Al cathode onto the HOMO of P3HT as long as electrons can be trapped in PC_71_BM near Al cathode, resulting in trap-assisted enhanced hole tunneling injection with EQE higher than 100%. An interesting phenomenon is that there is a distinct dip from about 490 nm to 570 nm and two apparent peaks at about 380 nm and 625 nm in the EQE spectra of the PM type PPDs. It is very apparent that EQE spectra of PM type PPDs can't match the absorption spectrum of P3HT, as shown in Figure 1c. The absorption spectra of blend films with different PC_71_BM doping weight ratios are shown in Figure S2. What is the underlying reason for the marked mismatch between the EQE and absorption spectra? The distinct dip from about 490 nm to 570 nm in the EQE spectra may be attributed to the weakened hole tunneling injection resulting from by fewer trapped electrons in PC_71_BM near Al cathode. The number of trapped electrons in PC_71_BM near Al cathode strongly depends on the optical field and exciton generation rate in this region. The optical field and the exciton generation rate distribution in the active layer of device A are simulated and shown in Figure S3. The simulated optical field intensity near the Al side is rather weak in the spectral range from 490 nm to 570 nm. The exciton generation rate is decreased due to the rather weak optical field intensity in this region, resulting in the fewer trapped electrons in PC_71_BM near Al cathode. Therefore, trap-assisted hole tunneling injection is weakened, leading to the relatively low EQE values in this spectral range.

In order to further demonstrate that the hole tunneling injection is assisted by trapped electrons in PC_71_BM near Al cathode, rather than inherent interfacial or bulk traps, the *J-V* characteristic curves of ITO/PEDOT:PSS/P3HT/LiF/Al device (neat P3HT as the active layer) were measured in dark and under 520 nm illumination with an intensity of 7.6 × 10^−6^ W cm^−2^, as shown in Figure S4. The *J-V* characteristic curves are almost entirely coincidence in dark and light conditions, which means that this device almost can't exhibit any photoresponse. Therefore, the observed PM phenomenon in devices A, B and C should be attributed to the hole tunneling injection assisted by trapped electrons in PC_71_BM near Al cathode.

A series of confirmatory devices with Al(1)/P3HT:PC_71_BM(100:1)/Al(2) device structure were purposely designed and fabricated on bare glass substrates to further explain the origin of the distinct dip in the EQE spectra of PM type PPDs. The only difference among these confirmatory devices is the thicknesses of the active layers adjusted from ~200 nm to ~350 nm by using various durations of rotation. The thicknesses of Al(1) and Al(2) layers are 16 nm and 100 nm, respectively. The relatively thin Al(1) layer was set as the anode and the light incident window. The barriers for hole injection from Al(1) and Al(2) onto the HOMO of P3HT are ~0.9 eV under 19 V and −19 V biases, respectively.

The EQE spectra of the confirmatory devices were measured under −19 V and 19 V biases and are shown in Figure 2a and 2b, respectively. It is apparent that EQE spectral shape of the confirmatory devices under reverse bias is very similar to those of devices A, B and C (as shown in Figure 1b). However, the peak EQE values of the confirmatory devices are much lower than that of device A, which is attributed to the rather low transmittance of Al(1) layer with a thickness of 16 nm (as shown in Figure S5). The EQE spectral shape of each confirmatory device exhibits significant difference under forward and reverse biases, especially in the strong absorption spectral range of P3HT. The different EQE spectral shape may be due to diverse widths of hole tunneling injection barrier adjusted by the number of trapped electrons in PC_71_BM near Al(1) or Al(2) cathodes. The simulated optical field and exciton generation rate distribution in the active layer of each confirmatory device are shown in Figure S6. According to the simulated optical field and exciton generation rate distribution in the active layer, it can be concluded that the number of trapped electrons in PC_71_BM near Al(1) cathode is larger than that near Al(2) cathode when the light is irridated from Al(1) cathode side. The energy levels of P3HT become more curved near the interface between P3HT and Al(1) cathode, which is induced by stronger Coulomb field built-up by more trapped electrons in PC_71_BM near Al(1) cathode, resulting in narrower hole injection barrier. The narrower interfacial barrier is beneficial to enhance hole tunelling injection for obtaining high EQE values. The spatial band diagrams of the confirmatory device under reverse and forward bias are shown in Figure 2c.

Another interesting phenomenon shown in Figure 2a and 2b is that EQE values of the confirmatory devices were decreased along with the increase of active layer thickness in the whole spectral range. Most importantly, the more significant EQE decrease trend along with the increase of active layer thickness is observed under reverse bias. The phenomenon can be well explained by following equation:
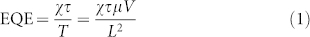
where *χ* is the fraction of excitons that dissociated into trapped electrons and free holes, *τ* is the lifetime of trapped electron, *T* is the hole transport time, *V* is the applied bias, *L* is the active layer thickness, and *μ* is the field dependent hole carrier mobility^26^. It is apparent that the hole transport time (*T*) is prelonged by the increase of active layer thickness (*L*), resulting in the decreased EQE values. The more signicifant EQE decrease trend along with the increase of active layer thickness under reverse bias (i.e., hole injection from Al(2) side) is attributed to the limited number of trapped electrons in PC_71_BM near Al(2) cathode due to the weakened optical field intensity and lower exciton generation rate in this region for light irridation from Al(1) side, as shown in Figure S6.

In order to further confirm the dependence of EQE values of all the PPDs on PC_71_BM doping weight ratio and wavelength of illumination light, the EQE-*V* characteristic curves were obtained under illumination with different wavelengths, as shown in Figure 3. It is apparent that the EQE values obtained in the EQE-*V* curves well accord with those obtained in the EQE spectra. The EQE values of PM type PPDs were increased along with the decrease of PC_71_BM doping ratios under the same reverse bias. The EQE values of photodiode type PPDs rapidly come into saturation (lower than 100%) under a small bias of ~−1.5 V. The EQE values of device E are slightly larger than those of device D at the same reverse bias, which should be attributed to the better exciton dissociation and charge carrier transport in the blend films with 1:1 P3HT:PC_71_BM weight ratio.

For devices A and B, the EQE values show exponential increase trend along with the increase of reverse bias, which accords with the voltage-dependent charge carrier injection phenomenon of Schottky contact^27^. Therefore, hole injection current assisted by trapped electrons in PC_71_BM near the Al cathode is dominating in the photoresponse current for devices A and B, which is distinct from the photoresponse current generated from photon-charge conversion process (i.e., photovoltaic process) in devices D and E. An extraordinary phenomenon observed in device C is that the EQE dependence on reverse bias shows a fast increase trend from 0 V to −5 V, a saturated trend from −5 V to −10 V and an exponential increase trend from −10 V to −19 V. It means that device C may work as a photodiode type PPD under low bias and as a PM type PPD under relatively high bias. The working mechanism of device C will be further clarified from the following transient photocurrent experiments. The detailed responsivity and EQE values of all the PPDs under specific wavelength light illumination are summarized and listed in Table 1, according to the EQE-*V* curves. The peak EQE value of device A is about 16,700% under −19 V bias and 380 nm illumination, corresponding to a responsivity of 51,700 mA W^−1^.

To further clarify the working mechanisms of the PPDs, the transient photocurrent of all the PPDs with different PC_71_BM doping weight ratios was measured under 625 nm light with an intensity of 9.17 × 10^−6^ W cm^−2^ modulated by an electronic shutter with a modulation period of 12 s, as shown in Figure 4. It is apparent that PM type PPDs (devices A, B and C) and photodiode type PPDs (devices D and E) show different response processes when the excitation light is turned on or turned off.

Devices A and B show a slow photoresponse process before arriving at saturation photocurrent when the excitation light is turned on. According to the above analysis, the photocurrent of devices A and B is mainly generated by hole tunneling injection assisted by trapped electrons in PC_71_BM near Al cathode. It should take some time to accumulate enough trapped electrons to arrive the dynamic balance between electron trapping process and detrapping processes, resulting in a relatively slow photoresponse process. This phenomenon, in turn, further proves that the photocurrent in devices A and B is generated by hole tunneling injection assisted by trapped electrons in PC_71_BM near Al cathode. The similar phenomenon can be observed from the transient photocurrent curves of device A under different illumination intensities, as shown in Figure S7. The transient photocurrent curves of devices A and B also show a slow decay process before arriving to the initial state (dark current) when the excitiation light is turned off, which corresponds to the slow release process of trapped electrons in PC_71_BM. The persistent photoconduction (slow decay process of photocurrent) after the light is turned off is due to the residual trapped electrons in PC_71_BM near Al cathode, which has been observed in previously reported photodetectors with high trap density^13^,^28^.

The transient photocurrent of photodiode type PPDs (devices D and E) exhibits an ultrafast rise or fall process when the excitation light is turned on or off. The ultrafast response of photodiode type PPDs can be explained by the photovoltaic mechanism without charge accumulation and release processes^4^,^29^. Device C presents slow or fast response processes under different biases, respectively. The fast rise or fall process is clearly observed under −7 V bias when excitation light is turned on or turned off. However, the slow response process is presented under −19 V bias. It once again proves that device C can work as a photodiode type PPD under low bias and as a PM type PPD under relatively high bias. The phenomenon observed from transient photocurrent curves of all the PPDs well accords with those obtained from their EQE spectra and EQE-*V* curves.

In order to better describe the working mechanism of PPDs with different PC_71_BM doping weight ratios, the schematic micro morphology of the active layers are shown in Figure 5. Much more isolated small PC_71_BM aggregations (i.e. electron traps) may be formed due to the low PC_71_BM doping weight ratio of 1% in the active layer, resulting in the hole tuneling injection assisted by more trapped electrons in PC_71_BM near Al cathode. The PC_71_BM aggregation trend is increased along with the increase of PC_71_BM doping weight ratio. It means that the fewer and larger PC_71_BM aggregations are formed in the active layers of devices B and C, resulting in the weakened hole tuneling injection.

Both photodiode and PM characterisitcs can be obsereved from device C under different bias, which can be well explained by PC_71_BM distribution in the active layer. Some PC_71_BM aggregations can connect to each other to form a few electron transport channels when PC_71_BM doping weight ratio is increased to 15%. Meanwhile, contact interface between PC_71_BM aggregations and Al cathode should be increased, resulting in more electrons transported to Al cathode at relatively low bias (exhibiting photodiode characteristics). For devices D and E, much more PC_71_BM aggregations will connect to each other to form better electron transport channels in the active layer. It is known that a bi-continuous interpenetrating network can be formed in the active layer with P3HT:PC_71_BM weight ratio of 1:1 (device E), which has been commonly reported in PSCs^30^,^31^. Therefore, devices D and E only show photodiode characteristics with limited EQE lower than unit.

For universal verification on this PM phenomenon induced by trap-assisted hole tunneling injection, PPDs with P3HT:PC_61_BM (100:1) as the active layer were fabricated with the same device structure as device A, exhibiting a peak EQE of about 13,500%, as shown in Figure S8. The relatively low EQE of 13,500% may be attributed to the relatively weak electron capturing ability of PC_61_BM molecule^24^.

In summary, we reported a novel type of solution-processed PPDs based on P3HT:PC_71_BM, which can exhibit photodiode and/or PM characteristics by only adjusting the PC_71_BM doping weight ratios. The maximum EQE of PM type PPDs is 16,700% for the device with PC_71_BM doping weight ratio of 1%. The PM phenomenon should be attributed to the enhanced hole tunneling injection assisted by trapped electrons in PC_71_BM near Al cathode. This discovery may open a new road for organic materials to be used in highly sensitive photodetectors while preserving the advantages of organic materials.

## Methods

### Preparation of solutions

P3HT (Product No: LT-S909, purchased from Luminescence Technology Corp) and PC_71_BM (Product No: LT-S923, purchased from Luminescence Technology Corp) were dissolved in 1,2-dichlorobenzene (extra pure, purchased from J&K Scientific Ltd.) to prepare 40 mg ml^−1^ solutions, respectively. Then, the P3HT and PC_71_BM solutions are blended in various volume ratios: 100:1, 100:4, 100:15, 100:50, and 1:1.

### Fabrication of PPDs

Indium tin oxides (ITO) coated glass substrates with a sheet resistance of 15 Ω square^−1^ (purchased from Shenzhen Jinghua Display Co., Ltd.) were pre-cleaned by ultrasonic treatment in detergent, deioned water and ethanol in ultrasonic sequentially. Then, all the substrates were dried by nitrogen-gas and treated by UV-ozone for 5 minutes to increase the work function of ITO. The solution of PEDOT:PSS (Clevios P VP. Al 4083, purchased from Heraeus Precious Metal Gmbh & Co. KG) was spin-coated onto the ITO glass substrates at 5000 rounds per minute (rpm) for 40 s. The PEDOT:PSS coated ITO glass substrates were baked in air at 120°C. After drying for 10 minutes, the substrates were transferred to a nitrogen-filled glove box (O_2_ and H_2_O concentrations < 1 p.p.m.). The solution of P3HT:PC_71_BM was spin-coated onto the PEDOT:PSS layers at 800 rpm for 30 s to prepare the organic active layers. The thickness of active layers is about 200 nm. The LiF interfacial layers of about 0.8 nm was deposited on the organic active layers, followed by the depositing of aluminum (Al) cathode layers of about 100 nm, through the shadow masks in vacuum (5 × 10^−4^ pa). The active area of each device is about 3.8 mm^2^, which is defined by the vertical overlap of the ITO anode and the Al cathode.

### Fabrication of confirmatory devices

The Al(1) layers of 16 nm were deposited onto the pre-cleaned glass substrates through the shadow masks in vacuum (5 × 10^−4^ pa). The solution of P3HT:PC_71_BM (100:1) was spin-coated onto the Al(1) layers at 800 rpm for various durations of rotation. Then, the Al(2) layers of about 100 nm were deposited on the active layers through the shadow masks in vacuum (5 × 10^−4^ pa). The active area is about 4 mm^2^, which is defined by the vertical overlap of the Al(1) and the Al(2) cathode. The confirmatory devices were fabricated in a nitrogen-filled glove box (O_2_ and H_2_O concentrations < 1 p.p.m.).

### Measurements

Current density versus voltage (*J–V*) curves of the PPDs were measured by a Keithley 2400 source meter in air conditions, under the 380 nm, 520 nm and 625 nm illuminations with intensities of 4.3 × 10^−6^ W cm^−2^, 7.6 × 10^−6^ W cm^−2^ and 5.8 × 10^−6^ W cm^−2^, respectively. Photocurrent was recorded using the Keithley 2400 Source Meter, while the wavelength of incident light was scanned from 300 nm to 700 nm. The monochromatic light used in all these measurements was provided by a 150 W xenon lamp coupled with a monochromator. The monochromatic light intensity and wavelength were monitored and calibrated by a Newport 818-UV power meter and an Acton SpectraPro 2150i CCD spectrometer. Absorption and transmittance spectra of films were measured by a Shimadzu UV-3101 PC spectrophotometer. The thickness of the Al(1) electrode layer was monitored by quartz monitor crystals and checked by an Ambios technology XP-2 stylus profilometer. The thicknesses of the active layers were measured by an Ambios technology XP-2 stylus profilometer.

### Calculations

EQE is calculated as
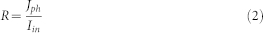

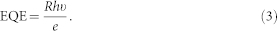
where *R* is the responsivity, *J_ph_* is the photocurrent density, *I_in_* is the intensity of incident light, *e* is absolute value of electron charge and *hυ* is the energy of incident photon, respectively.

## Author Contributions

F.Z. and L.L. conceived and designed the experiments. L.L., J.W., Q.A., Q.S. and W.W. performed the experiments. F.Z., L.L., J.W., Q.A., Q.S. and W.W. analyzed the data. F.Z. contributed materials and analysis tools. F.Z., L.L., J.Z. and F.T. wrote the paper. All authors reviewed the manuscript.

## Supplementary Material

Supplementary Information Supplementary Information

## Figures and Tables

**Figure 1:**
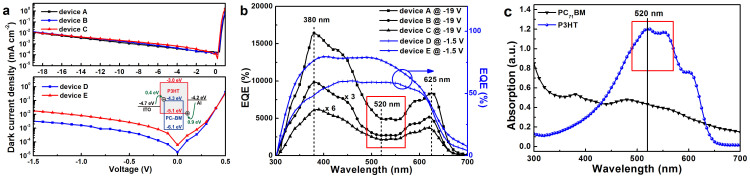
(a), Dark current density versus voltage (*J_d_-V*) curves, the inserted image is the energy levels of the used materials. (b), EQE spectra of the PPDs with different PC_71_BM doping weight ratios. (c), Absorption spectra of neat P3HT and PC_71_BM films.

**Figure 2:**
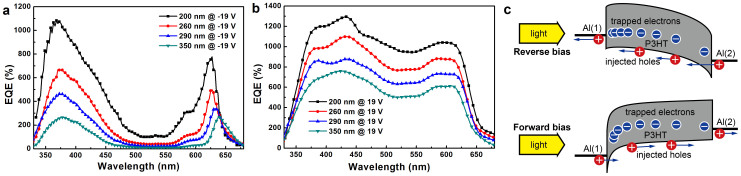
The EQE spectra of the Al(1)/P3HT:PC_71_BM(100:1)/Al(2) devices with different thicknesses of the active layers, under −19 V (a) and 19 V (b) biases, respectively. (c), The spatial band diagrams of Al(1)/P3HT:PC_71_BM(100:1)/Al(2) device under reverse and forward bias, respectively.

**Figure 3:**
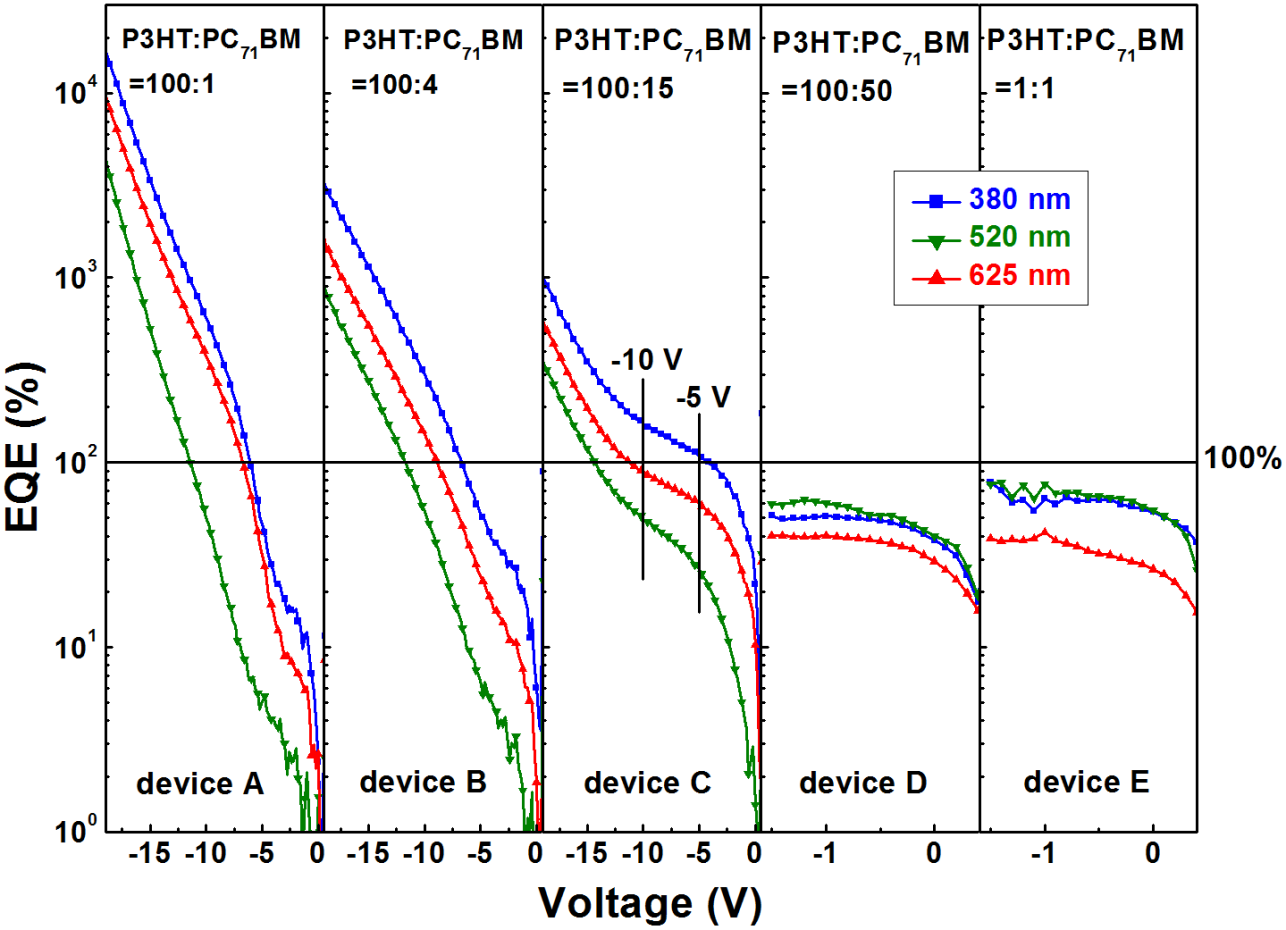
The EQE-*V* curves of all the PPDs under illumination with different wavelengths.

**Figure 4:**
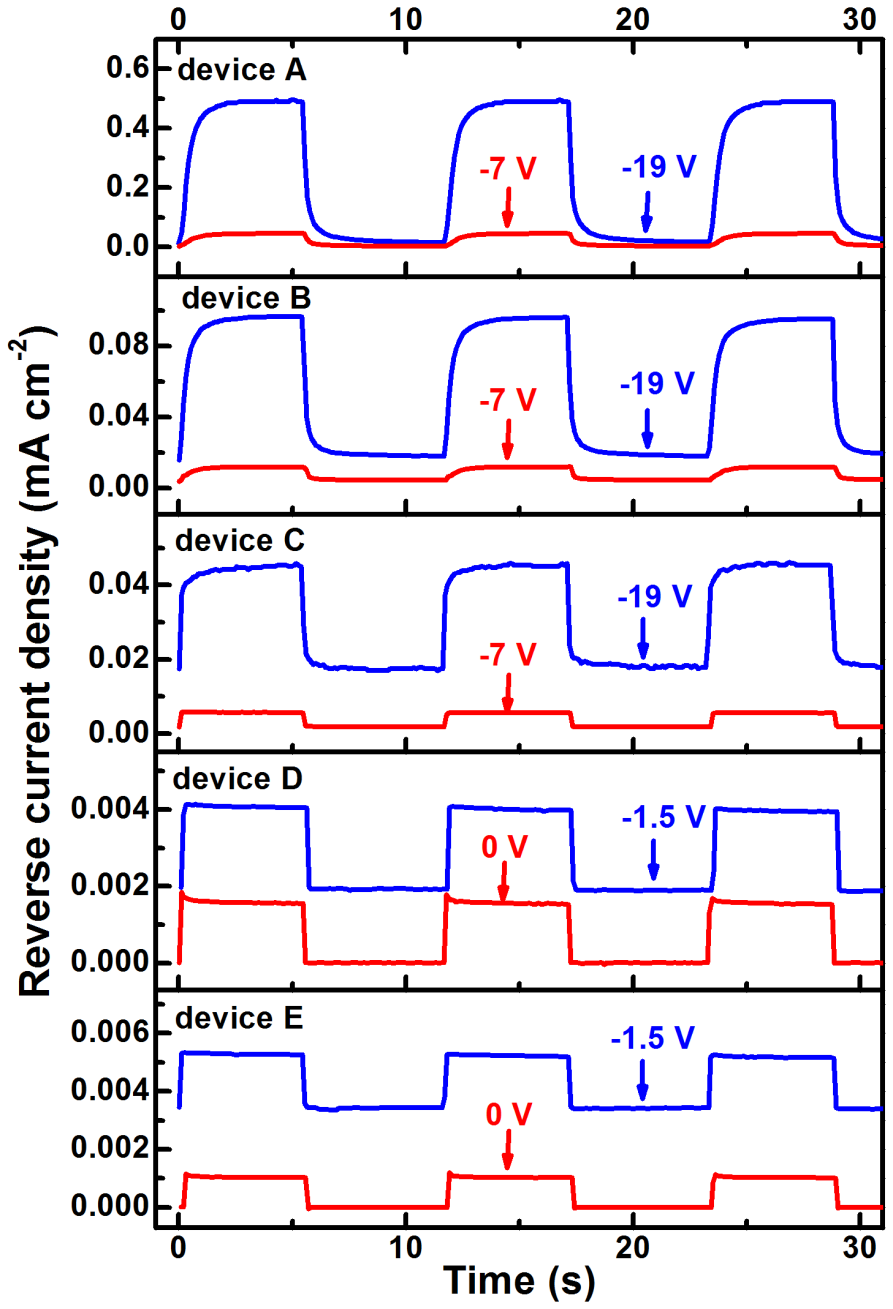
Transient photocurrent curves of all the PPDs under different biases. The 625 nm light source with an intensity of 9.17 × 10^−6^ W cm^−2^ was modulated by a electronic shutter with an modulation period of 12 s.

**Figure 5:**
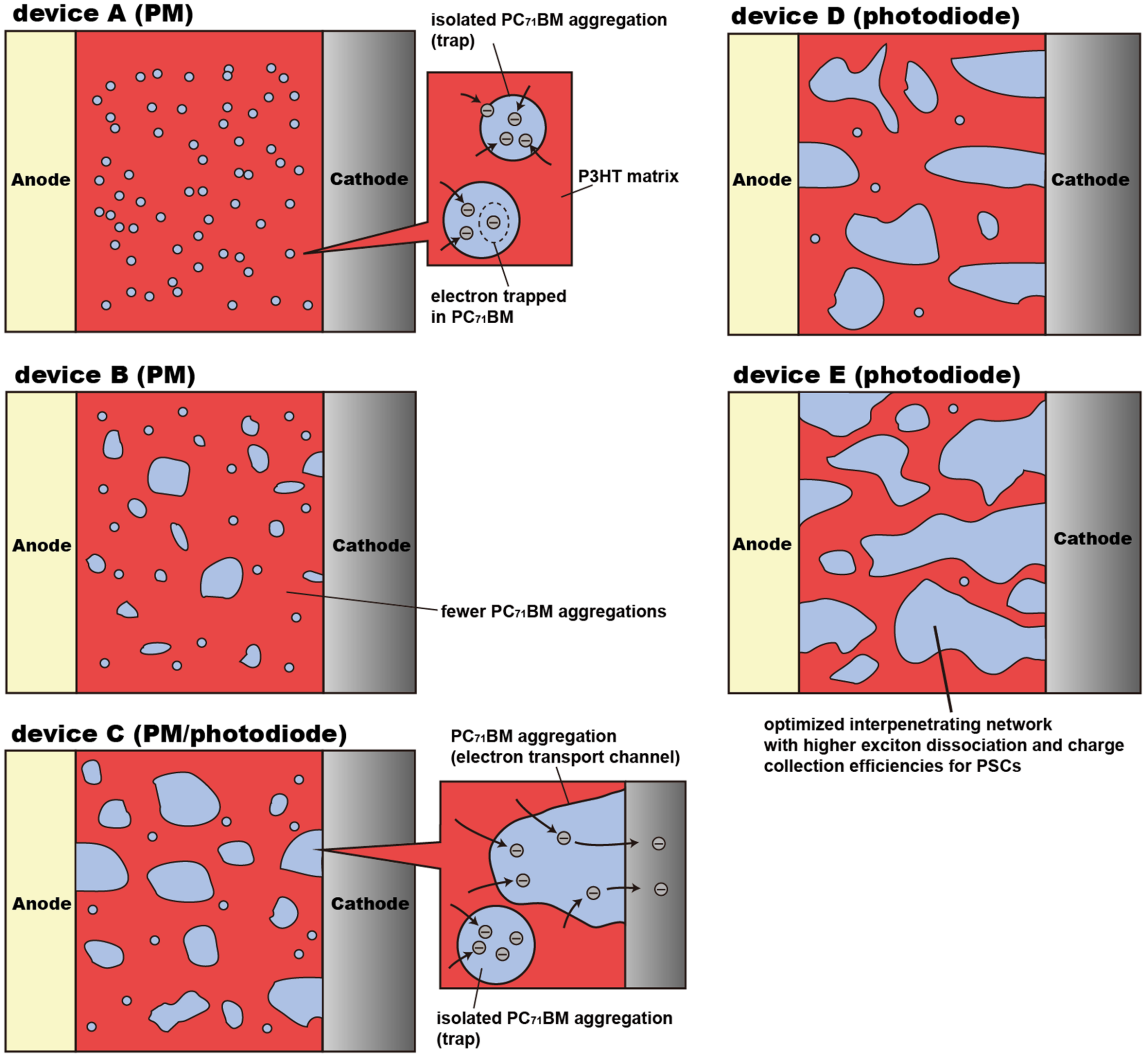
Schematic micro morphologies of active layers with different PC_71_BM doping weight ratios, corresponding to the PPDs from A to E.

**Table 1:** The P3HT:PC_71_BM weight ratios, mechanisms, operating biases, responsivities and EQE values of PPDs from A to E, respectively

P3HT:PC_71_BM weight ratio	100:1 (device A)	100:4 (device B)	100:15 (device C)	100:50 (device D)	100:100 (device E)
**Mechanism**	PMa)	PM	PM/Pb)	P	P
**Operating bias (V)**	–19	–19	–19	–1.5	–1.5
***R***c)** @ 380 nm (mA W^−1^)**	51,700	10,000	3,140	158	240
***R* @ 520 nm (mA W^−1^)**	18,600	3,800	1,490	250	320
***R* @ 625 nm (mA W^−1^)**	48,300	8,390	2,960	202	195
**EQE**d)** @ 380 nm (%)**	16,700	3,230	1,010	50.9	77.2
**EQE @ 520 nm (%)**	4,440	907	356	59.6	76.4
**EQE @ 625 nm (%)**	9,610	1,670	588	40.2	38.8

^a)^photomultiplication. ^b)^photodiode. ^c)^responsivity. ^d)^external quantum efficiency.
