# Supplementary material for: Achieving EQE of 16,700% in P3HT:PC71BM based photodetectors by trap-assisted photomultiplication
Source: Sci Rep. 2015 Mar 17;5:9181. doi: 10.1038/srep09181 (PMC4361887; doi:10.1038/srep09181)
Supplement: Supplementary Information [file srep09181-s1.pdf]

Supplementary Information for "Achieving EQE of 16,700% in P3HT:PC_{71}BM based photodetectors by trap-assisted photomultiplication"

Lingliang Li[1], Fujun Zhang[1], Jian Wang[1], Qiaoshi An[1], Qianqian Sun[1], Wenbin Wang[1], Jian Zhang[2], Feng Teng[1]*

1. Key Laboratory of Luminescence and Optical Information, Ministry of Education, Beijing Jiaotong University, Beijing 100044, People's Republic of China
2. State Key Laboratory of Catalysis, Dalian institute of Chemical Physics, Chinese Academy of Sciences, Dalian 116023, People's Republic of China

*Corresponding author e-mail: fjzhang@bjtu.edu.cn

For better understanding of the previous works on PM phenomenon based on organic or organic/inorganic hybrid materials, the schematic micro morphology of active layers for previous reported PM photodetectors are shown in **Figure S1**.

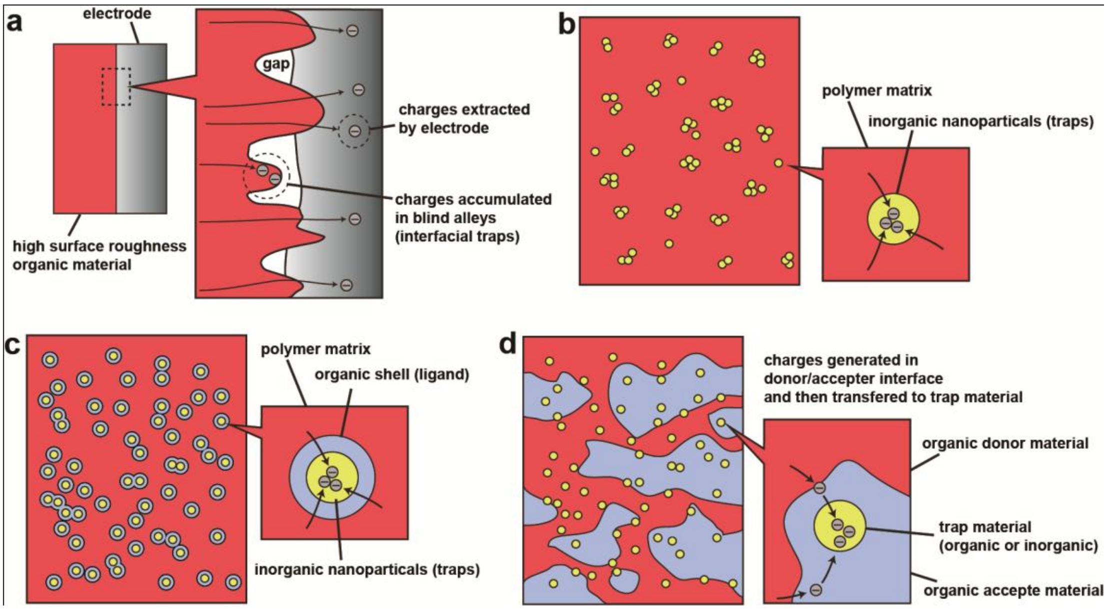

Figure S1 | Schematic micro morphology of active layers for several kinds of PM photodetectors based on organic materials: **a**, interfacial traps due to surface roughness [1-6]; **b**, polymer matrix with inorganic nanoparticles [7, 8]; **c**, polymer matrix with organic ligand coated nanoparticles (colloidal quantum dots) [9, 10]; **d**, donor/accepter system with additionally doped trap material [11-14].

The absorption spectra of neat P3HT and $PC_{71}BM$ films as well as the blend films with different $PC_{71}BM$ doping weight ratios are shown in **Figure S2**. It can be seen that the absorption spectra of the blend films are the superposition of the absorption spectra of neat P3HT and neat $PC_{71}BM$.

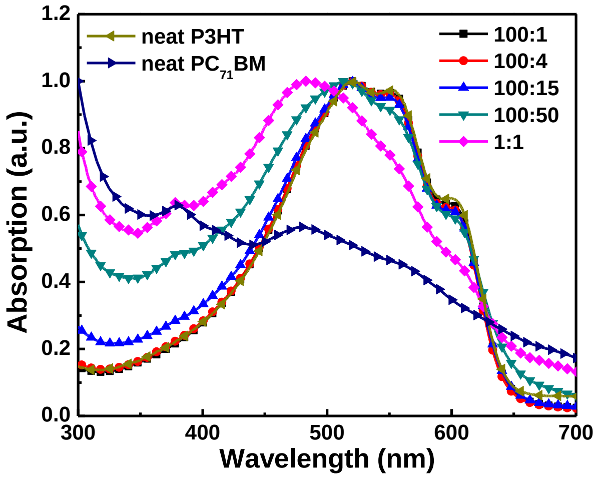

Figure S2 | The absorption spectra of neat P3HT and $PC_{71}BM$ films and the blend films with different $PC_{71}BM$ doping weight ratios.

The optical field distribution in the active layer of device A is simulated and shown in **Figure S3a**. It is apparent that the optical field intensity near the Al side in the wavelength range from 490 nm to 570 nm (i.e., the strong absorption wavelength range of P3HT) is much lower than that in other wavelength range. The rapid decay of the incident optical field intensity along with the propagation in the active layer should be attributed to the strong absorption of P3HT in this wavelength range. In other wavelength range, the decay of incident light intensity is much slow along with light propagation in the active layer, resulting in a distinct interferometry fringes formed between the incident light and reflected light.

In order to investigated the influence of optical field distribution on the exciton generation rate in the active layer of PM type PPDs, the corresponding exciton generation rate as a function of the

light propagation distance (position in active layer) in device A is simulated and shown in **Figure S3b**.

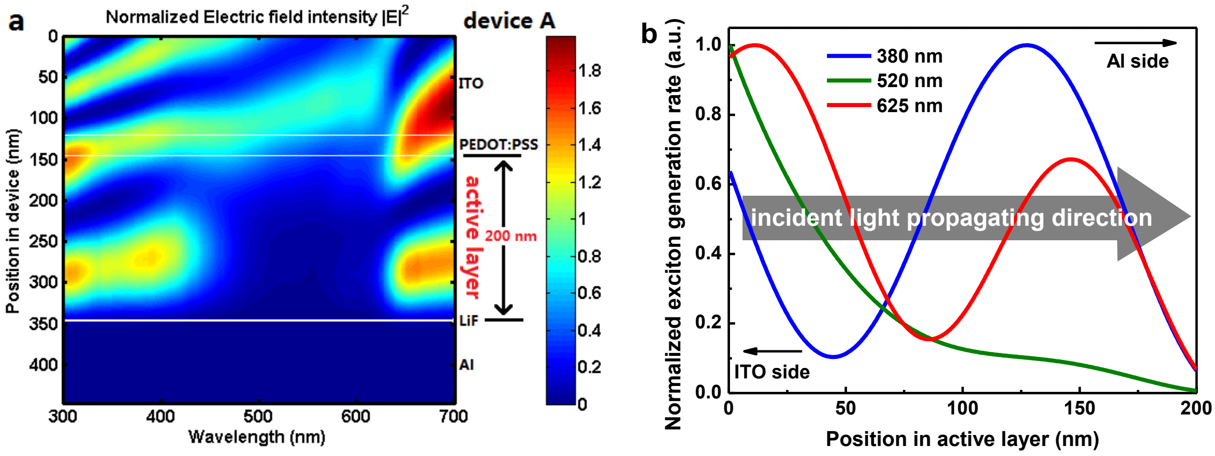

Figure S3 | a, The simulated optical field distribution induced by the incident light in device A. **b,** the corresponding exciton generation rate as a function of the light propagation distance (position in active layer) in device A.

For 520 nm light, it is apparent that the exciton generation rate is relatively high near the ITO anode side due to the strong absorption of P3HT at 520 nm. A distinct reduce trend of the exciton generation rate along with the increase of the light propagation distance is observed due to the decay of optical field distribution. Therefore, the exciton generation rate near the Al cathode side is rather low, resulting in the limited number of trapped electrons in $PC_{71}BM$ and weaken hole tunnelling injection assisted by the limited trapped electrons in $PC_{71}BM$ near the Al cathode side. The simulated results can well explian the distinct dip of the EQE spectrum for device A in the strong absorption spectral range of P3HT. As for 380 nm and 625 nm incident light, the exciton generation rate versus light propagation distance (position in active layer) curves well accords with the optical field distribution. The peaks of exciton generation rate in the curves should be attributed to the enhanced interferometry between incident light and reflected light from Al cathode.

In order to further demonstrate that the enhanced hole tunneling injection is assisted by trapped electrons in $PC_{71}BM$ near Al cathode, rather than inherent interfacial or bulk traps, the *J*-*V* characteristic curves of ITO/PEDOT:PSS/P3HT/LiF/Al device (neat P3HT as the active layer) were measured in dark and under 520 nm illumination with an intensity of 7.6×10^{-6} W cm^{-2}, as shown in **Figure S4**. The dark and light *J*-*V* characteristic curves are almost entirely coincidence, which means that this device almost can't exhibit any photoresponse. Therefore, the observed PM phenomenon in device A, B and C should be attributed to the enhanced hole tunneling injection assisted by trapped electrons in $PC_{71}BM$ near Al cathode.

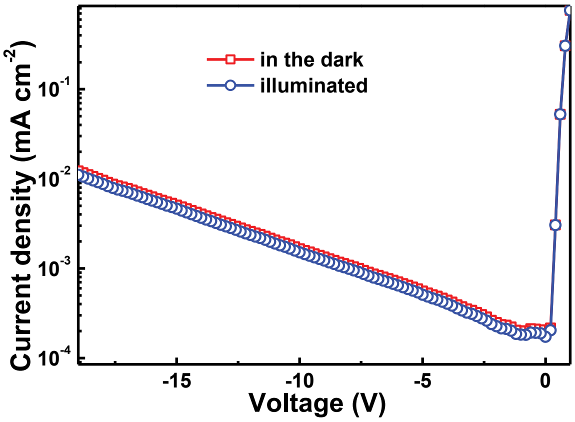

Figure S4 | The *J*-*V* curves of ITO/PEDOT:PSS/P3HT/LiF/Al device in dark and under 520 nm illumination with an intensity of 7.6×10^{-6} W cm^{-2}.

The transmittance spectra of the Al(1) coated glass substrate and ITO coated glass substrate are shown in **Figure S5**. It is apparent that the transmittance of Al(1) coated glass substrate is much lower than that of ITO coated glass substrate, resulting in the lower EQE values for the confirmatory devices in the whole spectral range compared with device A under the same reverse bias.

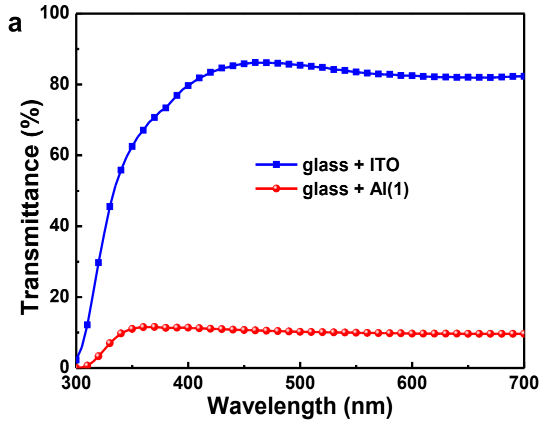

Figure S5 | Transmittance spectra of glass substrates coated with 16 nm Al and120 nm ITO, respectively.

The optical field distributions induced by the incident light in the confirmatory devices are simulated and shown in **Figure S6a to S6d**. The exciton generation rate as a function of the light propagation distance (position in active layer) in confirmatory devices with different thickness of active layer is culculated according to the optical field distribution, as shown in **Figure S6e to S6h**. It is apparent that the 520 nm light generated exciton generation rate near the Al(2) side is rapidly decreased along with the light propagation distance in the active layers, resulting in the limited number of trapped electrons in $PC_{71}BM$ near Al(2) side under reverse bias.The limited number of trapped electrons leads to the weaken hole tunneling injection assisted by trapped electrons. It can well explain the distinct dip in the EQE spectra in the strong absorption range of P3HT under reverse bias. For the device working at forward bias, the EQE values in whole spectral range are decreased along the increase of active layer thickness due to the longer hole transport time in the thicker active layer. The EQE spectral shape is almost kept constant for all device with different thickess of active layers. It can be explained from the exciton generation rate near Al(1) for light irridant from Al(1) side. Therefore, the variation of the EQE spectral shape for the confirmatory devices under reverse and forward biases should be attributed to the different hole tunneling

injection currents originated from different numbers of trapped electrons near the injection interface.

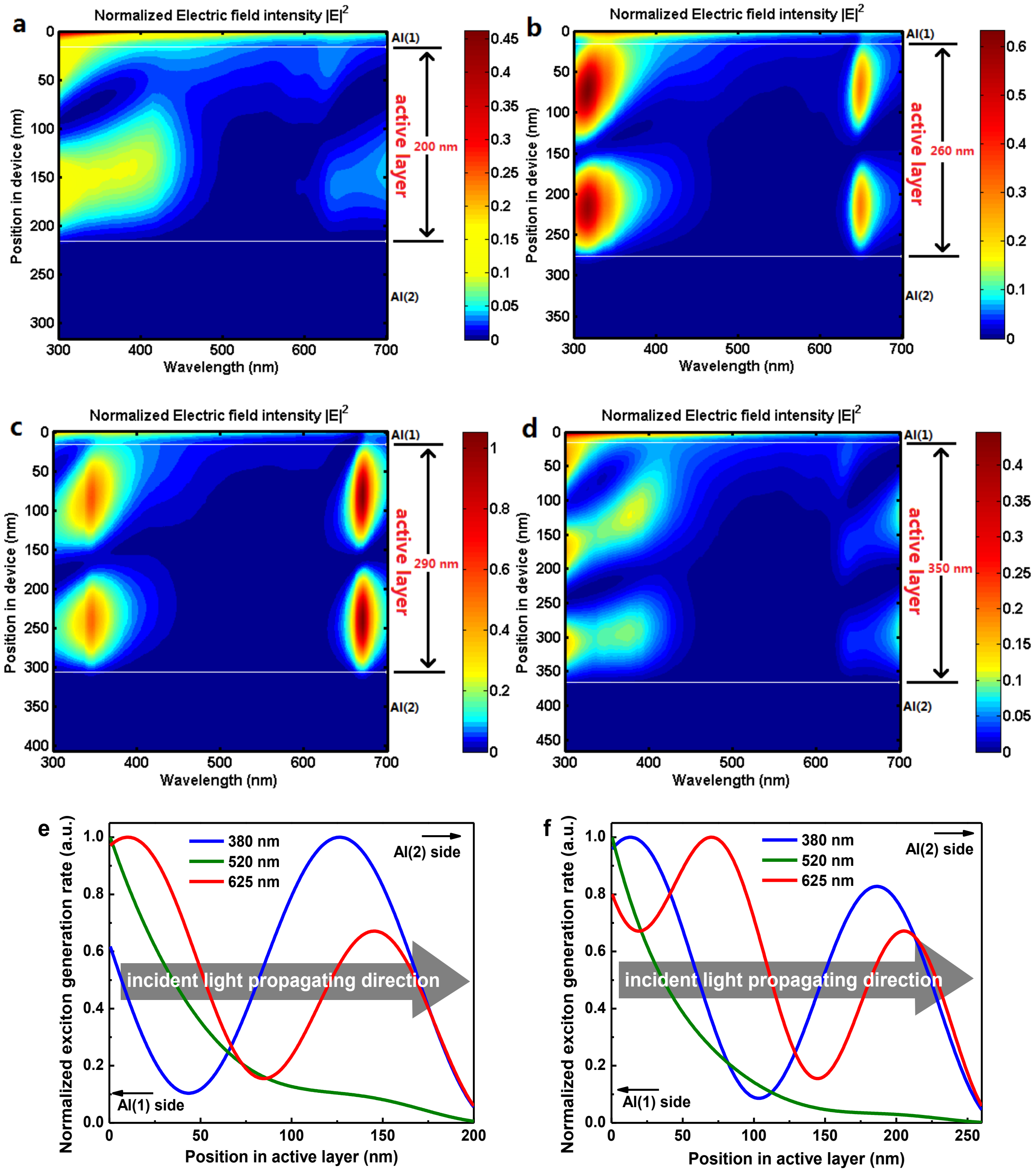

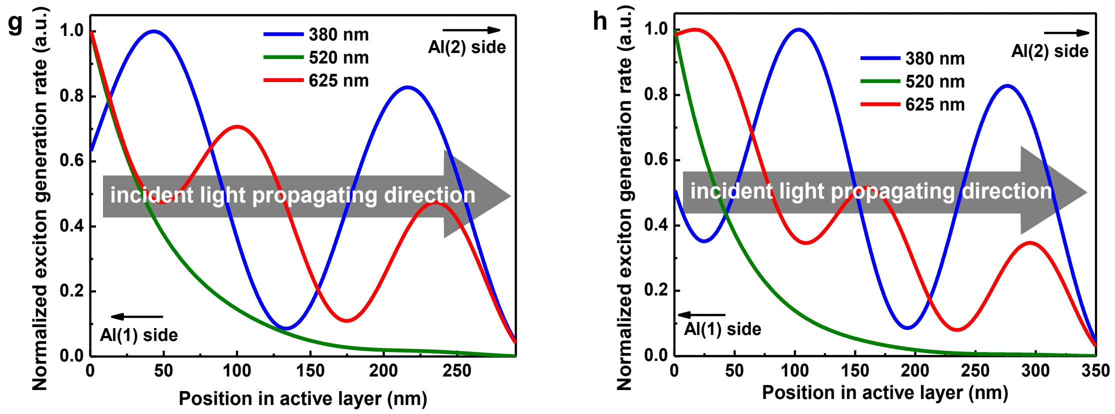

Figure S6 | a-d, The simulated optical field distributions of the incident light in the confirmatory devices with 200 nm (**a**), 260 nm (**b**), 290 nm (**c**) and 350 nm (**d**) active layers. **e-h,** the corresponding exciton generation rates as functions of the propagation distance of 380 nm, 520 nm and 625 nm light in confirmatory devices with 200 nm (**e**), 260 nm (**f**), 290 nm (**g**) and 350 nm (**h**) active layers.

Transient photocurrent of device A was measured under 625 nm light modulated by an electronic shutter with a modulation period of 12 s, as shown in **Figure S7.** Device A show a similar slow response characteristic under modulated light with different intensities of 9.17×10^{-6} W cm^{-2} and 3.85×10^{-6} W cm^{-2}. The saturated photocurrent under 9.17×10^{-6} W cm^{-2} light is higher than that under 3.85×10^{-6} W cm^{-2} light, which means that more trapped electrons will be accumulated in $PC_{71}BM$ near Al cathode under the stronger light intensity condition. It is interesting that the end of falling edge for the transient photocurrent under 9.17×10^{-6} W cm^{-2} light is almost coincident with that under 3.85×10^{-6} W cm^{-2}. It can be explained by the following model. The trapped electrons in $PC_{71}BM$ will be released when the excitation light is turn off, resulting in the weakened hole tunneling injection dependence on the number of trapped electrons in $PC_{71}BM$ near Al cathode. The transient photocurrent strongly depends on the trap-assisted hole tunneling injection. The decay process of transient photocurrent may be the same when the number of trapped electrons in $PC_{71}BM$ near Al cathode arrives to a certain level for any decay process. This phenomenon once again proves that PM device A is due to trap-assisted hole tunneling injection.

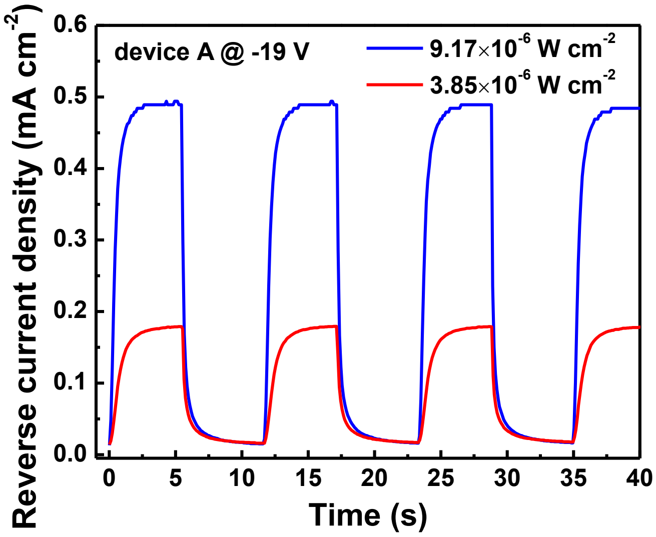

Figure S7 | The transient photocurrent density of device A under -19 V bias.

For universal verification on this PM phenomenon induced by trap-assisted hole tunneling injection, PPDs with P3HT:$PC_{61}BM$ (100:1) as the active layer were fabricated with the same device structure compared with device A, exhibiting a peak EQE of about 13,500%, as shown in **Figure S8**. The EQE spectral shape of devices with P3HT:$PC_{61}BM$ (100:1) as the active layer is very similar to that of device A under -19 V bias, which means the device with P3HT:$PC_{61}BM$ (100:1) as the active layer is also PM type PPD with hole tunneling injection assisted by trapped electrons in $PC_{61}BM$ aggregation near the Al cathode. The relatively low EQE of 13,500% for P3HT:$PC_{61}BM$ compared with that of 16,700% for P3HT:$PC_{71}BM$ may be attributed to the relatively weak electron capturing ability of $PC_{61}BM$ molecule.

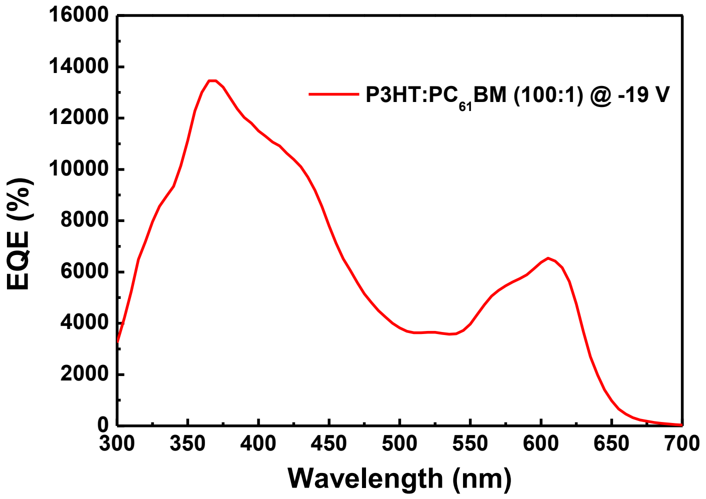

Figure S8 | The EQE spectrum of PPD (ITO/PEDOT:PSS/P3HT:PC_{61}BM/LiF/Al) under -19 V bias.

References:

[1] M. Hiramoto, T. Imahigashi, M. Yokoyama, Applied Physics Letters 64 (1994) 187-189.

[2] T. Katsume, M. Hiramoto, M. Yokoyama, Applied Physics Letters 69 (1996) 3722-3724.

[3] M. Hiramoto, A. Miki, M. Yoshida, M. Yokoyama, Applied Physics Letters 81 (2002) 1500-1502.

[4] J. Huang, Y. Yang, Applied Physics Letters 91 (2007) 203505.

[5] F. Guo, Z. Xiao, J. Huang, Advanced Optical Materials 1 (2013) 289-294.

[6] T.K. Däubler, D. Neher, H. Rost, H.H. Hörhold, Physical Review B 59 (1999) 1964-1972.

[7] F.W. Guo, B. Yang, Y.B. Yuan, Z.G. Xiao, Q.F. Dong, Y. Bi, J.S. Huang, Nature Nanotechnology 7 (2012) 798-802.

[8] I.H. Campbell, B.K. Crone, Journal of Applied Physics 101 (2007) 024502.

[9] G. Konstantatos, L. Levina, J. Tang, E.H. Sargent, Nano Letters 8 (2008) 4002-4006.

[10] D. Qi, M. Fischbein, M. Drndić, S. Šelmić, Applied Physics Letters 86 (2005) 093103.

[11] H.Y. Chen, M.K.F. Lo, G.W. Yang, H.G. Monbouquette, Y. Yang, Nature Nanotechnology 3 (2008) 543-547.

[12] S.T. Chuang, S.C. Chien, F.C. Chen, Applied Physics Letters 100 (2012) 013309.

[13] F.C. Chen, S.C. Chien, G.L. Cious, Applied Physics Letters 97 (2010) 103301.

[14] R. Dong, C. Bi, Q.F. Dong, F.W. Guo, Y.B. Yuan, Y.J. Fang, Z.G. Xiao, J.S. Huang, Advanced Optical Materials 2 (2014) 549-554.

[15] Y. Li, H.H. Huang, M.J. Wang, W.Y. Nie, W.X. Huang, G.J. Fang, D.L. Carroll, Solar Energy Materials And Solar Cells 98 (2012) 273-276.

[16] Y. Li, Three Dimensional Solar Cells based on Optical Confinement Geometries, Springer, USA, 2012. ISBN-13: 978-1461456988

[17] Y. Li, Open Photovoltaic Analysis Platform (OPVAP). http://www.opvap.com
